# Characteristics of Intraoperative Hemodynamic Instability in Postoperatively Diagnosed Pheochromocytoma and Sympathetic Paraganglioma Patients

**DOI:** 10.3389/fendo.2022.816833

**Published:** 2022-02-24

**Authors:** Jung Hee Kim, Hyung-Chul Lee, Su-jin Kim, Kyu Eun Lee, Kyeong Cheon Jung

**Affiliations:** ^1^Department of Internal Medicine, Seoul National University Hospital, Seoul National University College of Medicine, Seoul, South Korea; ^2^Department of Anesthesiology and Pain Medicine, Seoul National University Hospital, Seoul National University College of Medicine, Seoul, South Korea; ^3^Department of Surgery, Seoul National University College of Medicine, Seoul, South Korea; ^4^Cancer Research Institute, Seoul National University Hospital, Seoul National University College of Medicine, Seoul, South Korea; ^5^Division of Surgery, Thyroid Center, Seoul National University Cancer Hospital, Seoul, South Korea; ^6^Medical Big Data Research Center, Institute of Medical and Biological Engineering, Seoul National University, Seoul, South Korea; ^7^Department of Pathology, Seoul National University College of Medicine, Seoul, South Korea

**Keywords:** haemodynamic instability, pheochromocytoma, paraganglioma, intraoperative hypotension, intraoperative hypertension

## Abstract

**Background:**

Despite an improved understanding of pheochromocytoma and extra-adrenal sympathetic parganglioma (PPGL), including diagnosis and management, some PPGLs are postoperatively diagnosed. Clinical characteristics and intraoperative haemodynamic instability (HI) in postoperatively diagnosed PPGL patients have been poorly defined. Thus, we investigated the clinical characteristics and HI in patients with postoperatively diagnosed PPGLs compared to patients with preoperatively diagnosed PPGLs.

**Methods:**

We obtained clinical and haemodynamic data from the electronic medical records of 256 patients with pathologically confirmed PPGLs at our institution from January 2005 to December 2019. We assessed the intraoperative HI (systolic blood pressure [SBP]>160 mmHg (min) or mean blood pressure [MBP]<60 mmHg (min)) over time.

**Results:**

Twenty-nine patients (11.3%) were diagnosed with PPGLs postoperatively. Hypertension (34.5% *vs.* 63.0%, *P*=0.006) and pheochromocytoma (17.2% *vs.* 81.1%, *P*<0.001) case rates were lower in postoperatively diagnosed patients than in preoperatively diagnosed patients. Preoperative SBP in the ward was similar between groups, but the use of α-blockers and β-blockers was more frequent in preoperatively diagnosed patients (89.0% *vs.* 3.4%, *P*<0.001; 36.3% *vs.* 6.9%, *P*=0.003). Considering intraoperative HI, postoperatively diagnosed patients demonstrated a similar percentage of time with SBP>160 mmHg (median [IQR]; 7.9% [2.5; 11.9] % *vs.* 4.6% [0.0; 11.9], *P*=0.088) but a significantly lower percentage of time with MBP<60 mmHg (0.0% [0.0; 3.0] *vs.* 5.6% [0.0, 12.6], *P*=0.002) compared with preoperatively diagnosed patients.

**Conclusions:**

Patients diagnosed with PPGLs postoperatively may have no further higher risk of intraoperative hypertension than those diagnosed preoperatively despite insufficient preoperative management for PPGLs. Further study will be needed to ascertain intrinsic tumour characteristics, and need for universal preoperative use of α- and β-blockers in PPGL patients postoperatively diagnosed or without typical symptoms related PPGLs.

## Introduction

Pheochromocytoma and paraganglioma (PPGL) are rare catecholamine-secreting tumours originating from chromaffin cells of the adrenal medulla and extra-adrenal paraganglia. The overall prevalence of PPGL has been reported to range from 0.2% to 0.6%, and the overall age-standardized incidence rate is 0.18 per 100,000 person-years in Korea (1). Typical presentations that occur due to the release of catecholamines include headache, sweating, palpitation, and hypertension (2). Excessive catecholamine release from PPGLs can induce life-threatening complications such as myocardial infarction, heart failure, cardiomyopathy, shock, arrhythmias, and stroke (3, 4). However, the clinical presentation is highly variable, from completely asymptomatic to life-threatening complications. Thus, some PPGLs are diagnosed postoperatively in pathologic reports. In these cases, the preoperative medical preparation for PPGLs can be insufficient.

Surgery is the treatment of choice for PPGL patients. However, surgery is considered challenging due to intraoperative haemodynamic instability (HI). Intraoperative HI is characterized by arrhythmias, abrupt increases in blood pressure (BP) during intubation, manipulation of the tumour, and decreases in BP after ligation of the adrenal vein (5). To prevent intraoperative HI in PPGL patients, adequate preoperative management, including α-adrenergic receptor blockers followed by β-adrenergic receptor blockers, is needed to normalize the BP and heart rate (HR). Although advances in tumour localization techniques, proper preoperative preparation before PPGL resection, and anaesthetic management have resulted in improved surgical outcomes (5), intraoperative HI remains common with a highly variable incidence from 8.7% to 67%, and predictive factors for intraoperative HI are poorly defined (6, 7).

The proportion of incidentally diagnosed PPGLs are increasing (8–10). Kopetschke et al. reported that 29.4% of PPGL patients diagnosed incidentally, and 10% of patients were present without clinical symptoms (9). The recent study using Eurocrine^®^, the European registry for endocrine tumours revealed that incidentaloma were present in 43.4% (239/551 patients) of pheochromocytoma patients, 11.8% (65/551 patients) of patients were diagnosed as pheochromocytoma after adrenalectomy (11).

Especially for postoperatively diagnosed PPGL patients, these patients were unable to have proper preoperative preparation to prevent intraoperative HI. With insufficient preoperative antihypertensive treatment, using α-adrenergic receptor blockers followed by β-adrenergic receptor blockers or untreated hypovolemia, intraoperative HI can be life-threatening (12).

However, previous studies focused on intraoperative HI in PPGL patients who underwent surgical resection for PPGLs after routine preoperative medical treatment for PPGLs. There is little information on intraoperative HI and clinicopathologic features in postoperatively diagnosed PPGL patients. Moreover, several studies have investigated possible risk factors for intraoperative HI, focusing on hypertensive crisis, which emphasizes the importance of optimal preoperative α-adrenergic receptor blocker use. However, recent reports have demonstrated that intraoperative hypotension also contributes to intraoperative HI (6, 7, 13, 14).

In the present study, we compared clinical features, tumour characteristics, preoperative management, and preoperative haemodynamics in preopratively and postoperatively diagnosed PPGL patients and investigated whether, compared with preoperatively diagnosed patients, postoperatively diagnosed PPGL patients who experienced unexpected resection of PPGLs without preoperative preparation are at risk for intraoperative HI. Furthermore, we analysed the clinicopathologic risk factors for intraoperative hypotension in PPGL patients.

## Materials and Methods

### Study Subjects

To assess the hemodynamic risk for postoperatively diagnosed PPGLs, we retrospectively reviewed the pathological reports and included cases with pathologically confirmed PPGLs in Seoul National University Hospital (Seoul, Korea) from January 2005 to December 2019 (n=315). Among them, we excluded cases with head and neck PPGLs (n=59), which were assumed to be parasympathetic and non-functioning Finally, we included 256 PPGL cases: 189 cases with pheochromocytomas and 67 cases with extra-adrenal sympathetic paragangliomas. Sympathetic paragangliomas were located in the retroperitoneum (55 cases) and gastrointestinal tract (11 cases).

The present study was approved by the Institutional Review Board of the Seoul National University Hospital (No. H-1801-010-911), and informed consent was waived because of the retrospective design.

### Preoperative Variables

We retrieved clinical and haemodynamic data from electrical medical records as follows: a) age at initial diagnosis, sex, and body mass index; b) comorbidities, such as diabetes mellitus, hypertension, coronary artery diseases, cerebrovascular diseases, and history of abdominal surgery; c) tumour characteristics, such as maximal tumour size and metastasis; d) preoperative haemodynamic parameters, including BP and HR; and e) preoperative administration of α- and β-adrenergic receptor blockers. Diabetes mellitus was defined as an HbA1c ≥ 6.5% or the use of any oral anti-diabetic drugs or insulin therapy. Hypertension was defined as SBP ≥ 140 mmHg and/or DBP ≥ 90 mmHg on repeated measurements or the use of antihypertensive medications except α-adrenergic receptor blockers. Subjects having a history of percutaneous coronary intervention, coronary artery bypass surgery, or unstable angina were considered to have coronary artery disease. If there were medical records of ischemic or haemorrhagic stroke adjudicated by doctors, cerebrovascular diseases were indicated. Metastatic tumours were identified as the presence of chromaffin cell tumours in the lung, kidney, bone, liver, spleen and distant lymph nodes at diagnosis. α-adrenergic receptor blockers included phenoxybenzamine, doxazocin and terazocin. β-adrenergic receptor blockers included propranolol, bisoprolol, carvedilol, nebivolol, atenolol, metoprolol, and sotalol.

PPGLs were diagnosed based on excess preoperative catecholamine levels and/or pathology postoperatively. Patients with preoperatively diagnosed PPGLs exhibited catecholamine excess with or without typical clinical features, whereas those with postoperatively diagnosed PPGLs underwent surgery without catecholamine tests and were diagnosed by pathology. Catecholamine excess was examined with serum fractionated metanephrine/normetanephrine or 24-hour urine catecholamine/fractionated metanephrine. Urinary catecholamines and serum fractionated metanephrines were measured by liquid chromatography-tandem mass spectrometry, and urinary fractionated metanephrines were measured by high performance liquid chromatography-electrochemical detection.

Anatomical computed tomography (CT) or magnetic resonance imaging (MRI) was performed to locate and evaluate PPGLs. Multiplicity and metastases were evaluated using functional imaging, such as ^123^I-metaiodobenzylguanidine (MIBG) scan or ^68^Ga-labelled DOTA-Tyr3 octreotide (DOTATOC) positron emission tomography/computed tomography (PET/CT). Metastasis was defined as the presence of chromaffin cells in nonchromaffin organs, such as the lung, kidney, liver, and bone.

### Preoperative Medications

In preoperatively diagnosed cases, α-adrenergic receptor blockers were prescribed to reach BP<130/80 mmHg in the supine position and SBP>90 mmHg in the upright position. β-Adrenergic receptor blockers were added to subjects with an HR between 60–80 bpm only after α-adrenergic receptor blocker administration. In preoperatively diagnosed cases, preoperative volume replacement, including a high-salt diet and saline infusion, was conducted to prevent intra- and postoperative hypotension.

### Surgical Techniques

Operations were performed under general anaesthesia. In preoperatively diagnosed cases, an arterial line was routinely inserted into the patient’s radial artery after anaesthesia induction for continuous arterial pressure monitoring. The operation approach (open *vs.* laparoscopic) was determined based on the possibility of malignancy, tumour size, relationship to adjacent organs, and previous history of abdominal surgery. For most benign tumours < 7 cm on preoperative CT imaging, laparoscopic adrenalectomy was preferred. Combined resection was defined as the resection of organs other than PPGLs.

### Haemodynamic Variables

Haemodynamic variables, including BP and HR, were collected from the electronic anaesthetic chart, which included vital signs with a 1-minute interval and averaged with a 5-minute window. The surgical duration and anaesthesia duration were also collected from electronic medical records.

The intraoperative hemodynamic instabilities were defined as the time and the proportion of the intraoperative hemodynamic parameters outside the target ranges (SBP < 160 mmHg, MBP > 60 mmHg, or HR > 100 bpm). Intraoperative hypotension was defined as one or more occurrences of MBP < 60 mmHg.

We also analysed previously reported BP instability indexes. The average real variability (ARV), median performance error (MDPE), median absolute performance error (MDAPE), and wobble. These variables were calculated using the following formulas (14–16):


$$\text{ARV }(\text{mmHg})=\frac{1}{N-1}\sum_{k=1}^{N-1}\left|SBP_{k+1}-SBP_{k}\right|$$

$$\text{MDPE (\%)}=\underset{k=1,2,\cdots ,N}{\text{median}}\left(\frac{SBP_{k}-SBP_{ward}}{SBP_{ward}}\right)$$

$$\text{MDAPE (\%)}=\underset{k=1,2,\cdots ,N}{\text{median}}\left(\left|\frac{SBP_{k}-SBP_{ward}}{SBP_{ward}}\right|\right)$$

$$\text{Wobble (\%)}=\underset{k=1,2,\cdots ,N}{\text{median}}\left(\left|\frac{SBP_{k}-SBP_{ward}}{SBP_{ward}}-MDPE\right|\right)$$

where N represents the number of the SBP during the operation, SBP_k_ represents the k-th 5-minute average of the SBP during the operation, and SBP_ward_ represents the median ward BP measured one day before the operation.

### Statistical Analysis

Data are expressed as the mean ± standard deviation or median (interquartile range, IQR) for continuous variables and frequency (percentage) for categorical variables. We analysed continuous variables with a Student’s t-test or the Mann-Whitney U-test, depending on whether the data were normally distributed. We compared categorical variables with the Pearson chi-square test, or Fisher’s exact test. All analyses were performed using R version 3.6.2 (R Foundation for Statistical Computing, Vienna, Austria), and *P <*0.05 was considered significant.

## Results

We included 256 patients (49.2% women, mean age of diagnosis 50.8 ± 15.1 years). **Table 1** presents the clinical characteristics of patients diagnosed postoperatively (n=29) and preoperatively (n=227). There were no significant differences in age, sex, or BMI between the two groups.

**Table 1:** Clinical characteristics of patients according to the time of diagnosis.

Variables	Postoperatively diagnosed	Preoperatively diagnosed	Total	*P*
	(N = 29)	(N = 227)	(N = 256)	
Age, years	55.5 ± 11.8	50.1 ± 15.4	50.8 ± 15.1	0.074
Female	13 (44.8%)	113 (49.8%)	126 (49.2%)	0.760
Height, cm	166.2 [153.9;171.4]	163.0 [158.4; 169.3]	163.1[158.0;169.6]	0.719
Body weight, kg	62.6 ± 9.2	62.3 ± 11.2	62.3 ± 11.0	0.875
BMI, kg/m^2^	23.7 [21.9;25.1]	22.9 [20.9;25.4]	23.2 [21.0; 25.3]	0.612
Diabetes mellitus	6 (20.7%)	58 (25.6%)	64 (25.0%)	0.733
**Hypertension**	**10 (34.5%)**	**143 (63.0%)**	**153 (59.8%)**	**0.006**
Coronary artery disease*	2 (6.9%)	27 (11.9%)	29 (11.3%)	0.548
Cerebrovascular disease*	0 (0.0%)	14 (6.2%)	14 (5.5%)	0.379
History of abdominal surgery	7 (24.1%)	74 (32.6%)	81 (31.6%)	0.477
**Pheochromocytoma**	**5 (17.2%)**	**184 (81.1%)**	**189 (73.8%)**	**<0.001**
Maximal tumour size, cm	4.0 [1.5; 6.5]	4.5 [2.7; 6.2]	4.5 [2.6; 6.3]	0.272
Multifocal*	2 (6.9%)	28 (12.3%)	30 (11.7%)	0.547
Metastasis*	0 (0.0%)	19 (8.4%)	19 (7.4%)	0.142
Preoperative ward vital sign				
Mean ward SBP, mmHg	121.2 [113.3;130.0]	117.7 [110.2;126.4]	118.5 [110.4;127.0]	0.117
Minimum ward SBP, mmHg	113.0 [103.0;121.0]	108.0 [100.0;116.0]	109.0 [100.5;118.0]	0.056
Maximum ward SBP, mmHg	130.0 [120.0;138.0]	129.0 [116.0;139.5]	129.0 [117.0;139.0]	0.499
α-blocker use*	**1 (3.4%)**	**202 (89.0%)**	**203 (79.3%)**	**< 0.001**
β-blocker use*	**2 (6.9%)**	**82 (36.3%)**	**84 (32.9%)**	**0.003**

Data are presented as the mean ± standard deviation, median (interquartile range), or number (percentages). BMI, body mass index; SBP, systolic blood pressure. *Fisher’s exact test was used for analysis.

The prevalence of hypertension was lower in postoperatively diagnosed patients than in preoperatively diagnosed patients, whereas other comorbidities were similar between the two groups. Regarding the tumour characteristics, the proportion of pheochromocytoma was lower in postoperatively diagnosed patients than in preoperatively diagnosed patients (17.2% *vs.* 81.1%, *P <*0.001). However, the maximal tumour size, multifocality and metastasis were not different between the two groups. Preoperative SBP in the ward was similar between groups, but the use of α-blockers and β-blockers was more frequent in preoperatively diagnosed patients to reduce severe hypertension and tachyarrhythmia during the operation. In postoperative diagnosed patients, α-blockers and β-blockers were prescribed for another purpose, such as benign prostate hyperplasia or antihypertensive medications.

**Figure 1:**
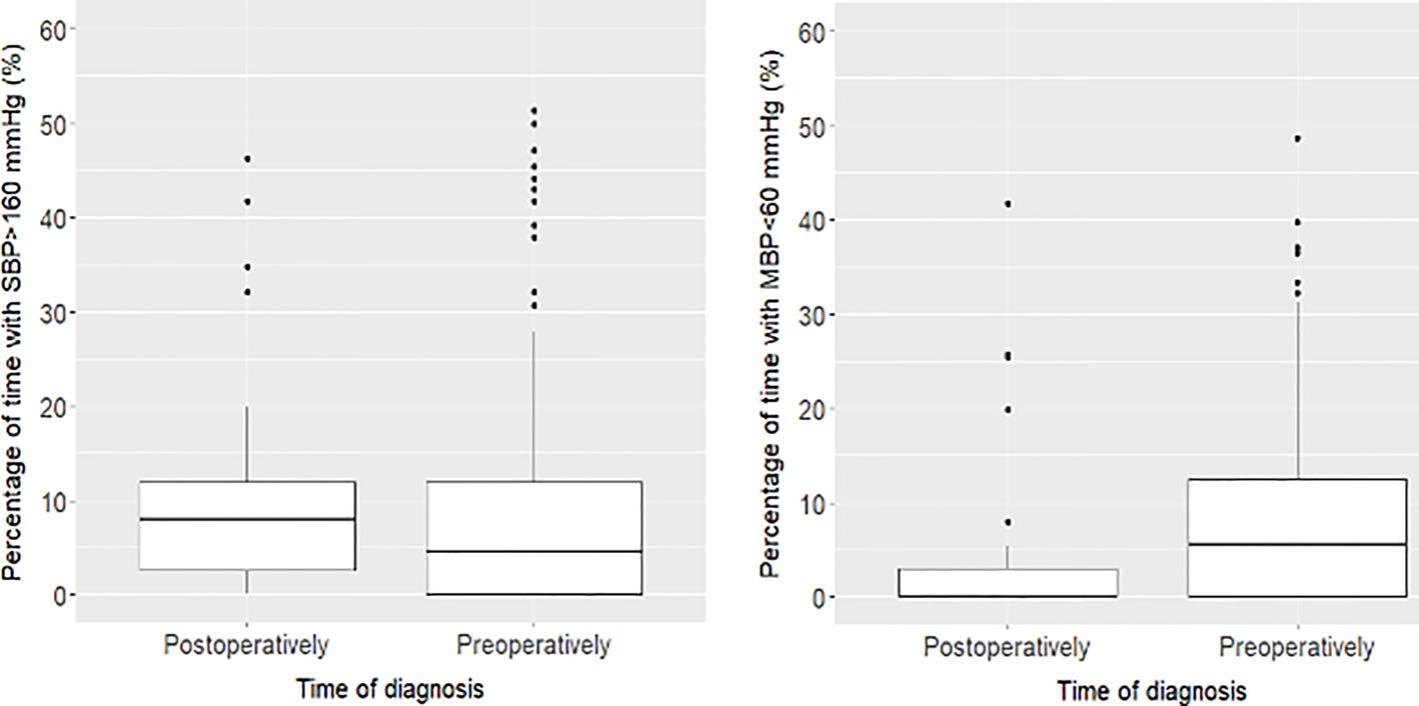
Percentage of time with BP outside the target ranges (SBP > 160 mmHg, or MBP < 60 mmHg) over surgical duration according to the time of diagnosis. Postoperatively diagnosed patients demonstrated a similar percentage of time with an SBP > 160 mmHg (median [IQR]; 7.9% [2.5; 11.9] % *vs.* 4.6% [0.0; 11.9], *P* = 0.088) but a significantly lower percentage of time with an MBP <60 mmHg (0.0% [0.0; 3.0] *vs.* 5.6% [0.0, 12.6], *P* = 0.002) compared with preoperatively diagnosed patients.

**Figure 1** illustrates the percentage of time with BP outside the target ranges (SBP > 160 mmHg, or MBP < 60 mmHg) according to the time of diagnosis. Postoperatively diagnosed patients demonstrated a similar percentage of time with an SBP > 160 mmHg (median [IQR]; 7.9% [2.5; 11.9] % *vs.* 4.6% [0.0; 11.9], *P* = 0.088) but a significantly lower percentage of time with an MBP < 60 mmHg (0.0% [0.0; 3.0] *vs.* 5.6% [0.0, 12.6], *P* = 0.002) compared with preoperatively diagnosed patients.

In **Table 2**, the median values of intraoperative SBP, MBP, and HR tended to be higher in postoperatively diagnosed patients than in preoperatively diagnosed patients. However, the maximum value of SBP was similar between the two groups, while the minimum value of MBP was significantly lower in preoperatively diagnosed patients. The time with an HR >100 bpm was not significant between the two groups. Other haemodynamic variables, such as ARV, MDPE, MDAPE, and wobble, were not different. Open surgery was more frequently performed in postoperatively diagnosed patients. and the duration of hospitalization was longer in postoperatively diagnosed patients. There was no 30-day mortality-related surgery in either group.

**Table 2:** Hemodynamic parameter of patients according to the time of diagnosis.

Variables	Postoperatively diagnosed	Preoperatively diagnosed	Total	*P*
	(N = 29)	(N = 227)	(N = 256)	
Intraoperative vital sign				
Mean SBP, mmHg	**123.8 [118.1;125.5]**	**116.0 [109.1;123.4]**	**116.8 [110.2;124.1]**	**0.001**
Minimum SBP, mmHg	78.0 [68.0;84.0]	74.0 [66.0;82.0]	74.0 [66.0;82.5]	0.237
Maximum SBP, mmHg	196.0 [178.0;210.0]	186.0 [167.5;208.0]	188.5 [168.5;209.0]	0.229
Mean MBP, mmHg	**90.2 [84.2;94.6]**	**82.9 [78.4;89.1]**	**83.6 [79.1;89.9]**	**< 0.001**
Minimum MBP, mmHg	**58.0 [52.0;62.0]**	**52.0 [46.0;58.0]**	**52.0 [46.5;58.0]**	**0.003**
Maximum MBP, mmHg	140.0 [131.0;163.5]	134.0 [121.5;154.5]	136.0 [122.0;156.0]	0.097
Mean HR, bpm	**72.8 [67.5;83.2]**	**68.8 [62.0;77.1]**	**70.1 [63.0;78.0]**	**0.018**
Minimum HR, bpm	**59.0 [52.0;64.0]**	**50.0 [44.0;56.0]**	**50.0 [44.0;58.0]**	**<0.001**
Maximum HR, bpm	107.0 [97.0;119.0]	102.0 [92.0;112.0]	104.0 [92.0;114.0]	0.139
Hemodynamic instability				
Time with SBP > 160 mmHg, min	10.0 [5.0; 20.0]	5.0 [0.0; 15.0]	5.0 [0.0; 15.0]	0.167
Percentage of time with SBP > 160 mmHg, %	7.9 [2.5; 11.9]	4.6 [0.0; 11.9]	4.9 [0.0; 11.9]	0.088
**Time with MBP < 60 mmHg, min**	**5.0 [0.0; 15.0]**	**20.0 [5.0; 50.0]**	**15.0 [5.0;50.0]**	**0.004**
**Percentage of time with MBP < 60 mmHg, %**	**0.0 [0.0; 3.0]**	**5.6 [0.0; 12.6]**	**4.9 [0.0; 11.9]**	**0.002**
Time with HR>100, min	5.0 [0.0; 7.5]	0.0 [0.0; 10.0]	0.0 [0.0; 10.0]	0.443
Percentage of time with HR>100, %	2.1 [0.0; 9.9]	0.0 [0.0; 7.3]	0.0 [0.0; 7.6]	0.295
ARV	12.0 [9.0;13.7]	9.6 [6.2;14.3]	10.0 [6.4;14.3]	0.177
MDPE	-1.0 [-7.4; 3.9]	-3.4 [-12.2; 4.0]	-2.8 [-12.1; 4.1]	0.388
MDAPE	14.2 [10.9;18.5]	15.1 [11.8;19.7]	15.0 [11.8;19.7]	0.514
Wobble	11.2 [8.1;16.6]	11.1 [8.3;14.3]	11.1 [8.3;14.4]	0.396
Anaesthesia duration, hour	3.2 [2.3;4.2]	3.1 [2.6;4.0]	3.1 [2.5;4.2]	0.749
Surgical duration, hour	2.3 [1.6;3.2]	2.1 [1.5;3.0]	2.1 [1.5;3.1]	0.498
Combined resection*	4 (13.8%)	12 (5.3%)	16 (6.2%)	0.092
Open surgery	27 (93.1%)	101 (44.7%)	128 (50.0%)	<0.001
Readmission rate*	1 (3.4%)	4 (1.8%)	5 (2.0%)	0.455
Hospitalization, days	6.0 [4.0; 8.0]	5.0 [3.0; 7.5]	5.0 [3.0; 8.0]	0.050

Data are presented as the mean ± standard deviation or median (interquartile range), or number (percentages).

SBP, systolic blood pressure; MBP, mean blood pressure; HR, heart rate; bpm, beats per minute; ARV, average real variability; MDPE, median performance error; MDAPE, median absolute performance error. *Fisher’s exact test was used for analysis.

We further compared the haemodynamic parameters only in patients with paragangliomas according to the time of diagnosis (**Table 3**). The use of α-blockers and β-blockers in patients diagnosed with paraganglioma preoperatively (n=43) was more frequent in those diagnosed postoperatively (n=24). The overall haemodynamic parameters did not differ between the two groups except that the mean MBP was higher in postoperatively diagnosed patients than in preoperatively diagnosed patients.

**Table 3:** Hemodynamic parameter of only patients with paraganglioma (PGL) according to the time of diagnosis.

Variables	Postoperatively diagnosed PGL	Preoperatively diagnosed PGL	Total	*P*
	(N = 24)	(N = 43)	(N = 67)	
Age, years	57.5 [47.5;61.5]	50.0 [34.0;62.5]	54.0 [43.0;62.0]	0.112
Female	11 (45.8%)	20 (46.5%)	31 (46.3%)	1.000
BMI, kg/m^2^	23.7 [22.0;24.6]	22.7 [20.8;23.9]	23.0 [21.2;24.1]	0.082
**Hypertension**	**9 (37.5%)**	**25 (58.1%)**	**34 (50.7%)**	**0.006**
Maximal tumour size, cm	4.5 [1.6; 7.0]	5.2 [3.1; 6.4]	5.0 [2.6; 6.5]	0.244
Multifocal	1 (4.2%)	7 (16.3%)	8 (11.9%)	0.242
Metastasis	0 (0.0%)	5 (11.6%)	5 (7.5%)	0.151
Preoperative ward vital sign				
Mean ward SBP, mmHg	122.8 [115.5;132.0]	120.6 [110.2;129.4]	120.8 [111.1;130.5]	0.601
Minimum ward SBP, mmHg	114.5 [104.0;122.0]	110.0 [100.5;119.0]	110.0 [102.5;120.0]	0.280
Maximum ward SBP, mmHg	132.5 [120.0;138.5]	129.0 [114.5;144.5]	131.0 [116.5;143.0]	0.773
α-blocker use*	**0 (0.0%)**	**31 (72.1%)**	**31 (46.3%)**	**< 0.001**
β-blocker use*	**1 (4.2%)**	**13 (30.2%)**	**14 (20.9%)**	**0.028**
Intraoperative vital sign				
Mean SBP, mmHg	122.4 [115.8;125.4]	120.0 [112.2;126.9]	121.0 [114.7;126.1]	0.307
Minimum SBP, mmHg	79.0 [67.0;84.0]	76.0 [67.5;87.5]	78.0 [67.5;86.0]	0.744
Maximum SBP, mmHg	189.0 [174.0;220.0]	185.0 [172.5;200.0]	188.0 [173.5;202.0]	0.556
**Mean MBP, mmHg**	**90.0 [84.4;93.3]**	**82.5 [79.0;88.6]**	**84.8 [81.0;91.4]**	**0.003**
Minimum MBP, mmHg	58.0 [53.0;62.0]	54.0 [48.0;60.0]	56.0 [50.0;60.5]	0.141
Maximum MBP, mmHg	139.0 [129.0;165.2]	135.0 [124.0;146.5]	137.0 [126.0;151.0]	0.363
Mean HR, bpm	72.2 [67.9;80.8]	74.8 [65.3;79.5]	73.9 [65.3;79.8]	0.686
Minimum HR, bpm	58.0 [52.0;64.0]	54.0 [48.0;61.0]	54.0 [50.0;62.0]	0.072
Maximum HR, bpm	104.0 [97.0;115.0]	104.0 [96.0;116.0]	104.0 [96.0;116.0]	0.743
Intraoperative hemodynamic instability				
Time with SBP > 160 mmHg, min	10.0 [2.5;22.5]	10.0 [0.0;15.0]	10.0 [0.0;17.5]	0.527
Percentage of time with SBP > 160 mmHg, %	7.9 [2.2;14.0]	6.4 [0.0;13.1]	6.4 [0.0;13.1]	0.447
Time with MBP < 60 mmHg, min	0.0 [0.0; 7.5]	5.0 [0.0;15.0]	0.0 [0.0;10.0]	0.171
Percentage of time with MBP < 60 mmHg, %	0.0 [0.0; 3.2]	3.2 [0.0; 8.6]	0.0 [0.0; 8.0]	0.172
Time with HR>100, min	0.0 [0.0; 5.0]	0.0 [0.0; 5.0]	0.0 [0.0; 5.0]	1.000
Percentage of time with HR>100, %	0.0 [0.0; 7.4]	0.0 [0.0; 8.3]	0.0 [0.0; 8.1]	0.955
ARV	12.2 [7.2;14.0]	7.9 [6.2;12.7]	10.6 [6.7;13.6]	0.165
MDPE	-1.3 [-12.1; 3.7]	-1.3 [-12.9; 9.2]	-1.3 [-12.4; 5.5]	0.963
MDAPE	16.6 [10.8;19.0]	14.3 [11.8;20.4]	14.8 [11.3;19.7]	0.784
Wobble	11.2 [7.8;17.5]	11.1 [8.6;14.3]	11.2 [8.3;14.6]	0.578
Anaesthesia duration, hour	3.2 [2.2; 4.2]	3.0 [2.4; 3.8]	3.2 [2.3; 4.2]	0.922
Surgical duration, hour	2.2 [1.4; 3.2]	2.2 [1.6; 2.9]	2.2 [1.5; 3.1]	0.675
Combined resection*	3 (12.5%)	2 (4.7%)	5 (7.5%)	0.341
Open surgery	23 (95.8%)	37 (86.0%)	60 (89.6%)	0.407
Readmission rate*	1 (4.2%)	2 (4.7%)	3 (4.5%)	1.000
Hospitalization, days	7.0 [4.0; 8.5]	6.0 [4.5; 9.0]	7.0 [4.0; 9.0]	0.664

Data are presented as the mean ± standard deviation or median (interquartile range), or number (percentages).

PGL, paraganglioma; SBP, systolic blood pressure; MBP, mean blood pressure; HR, heart rate; bpm, beats per minute; ARV, average real variability; MDPE, median performance error; MDAPE, median absolute performance error. *Fisher’s exact test was used for analysis.

Next, we compared the clinical characteristics of patients according to the occurrence of intraoperative hypotension defined as an MBP<60 mmHg (**Table 4**). Postoperatively diagnosed patients were less likely to experience intraoperative hypotension. The occurrence of intraoperative hypotension was related to larger tumour size, α-blocker use, lower ward SBP, and longer surgical duration.

**Table 4:** Clinical characteristics of patients according to the occurrence of intraoperative hypotension (intraoperative mean blood pressure (MBP) <60 mmHg).

Variables	Intraoperative hypotension (-)	Intraoperative hypotension (+)	*P*
	(n = 93)	(N = 163)	
**Postoperatively diagnosed**	**19 (20.4%)**	**10 (6.1%)**	**0.001**
Age, years	52.3 ± 13.6	49.9 ± 15.9	0.214
Female	17 (41.5%)	109 (50.7%)	0.361
Height, cm	163.5 [158.2; 169.9]	163.0 [157.8; 169.2]	0.574
Body weight, kg	63.3 ± 11.8	61.8 ± 10.5	0.275
BMI, kg/m^2^	23.4 [21.2; 25.8]	22.8 [21.0; 25.2]	0.388
Diabetes	21 (22.6%)	43 (26.4%)	0.599
Hypertension	51 (54.8%)	102 (62.6%)	0.279
Coronary artery disease	10 (10.8%)	19 (11.7%)	0.988
Cerebrovascular disease*	3 (3.2%)	11 (6.7%)	0.365
History of abdominal surgery	28 (30.1%)	53 (32.5%)	0.796
**Pheochromocytoma**	**57 (61.3%)**	**132 (81.0%)**	**0.064**
**Maximal tumour size, cm**	**3.8 [2.1; 5.2]**	**4.8 [3.2; 6.7]**	**<0.001**
Multifocal	14 (15.1%)	16 (9.8%)	0.293
Metastasis	7 (7.5%)	12 (7.4%)	1.000
**α-blocker use**	**63 (67.7%)**	**140 (85.9%)**	**0.001**
β-blocker use	30 (32.3%)	54 (33.3%)	0.970
Preoperative ward vital sign			
Mean ward SBP, mmHg	120.5 [113.3; 127.5]	117.4 [109.4; 126.8]	0.065
**Minimum ward SBP, mmHg**	**111.0 [103.0; 119.0]**	**106.0 [100.0; 115.5]**	**0.019**
Maximum ward SBP, mmHg	131.0 [121.0; 140.0]	128.0 [116.0; 139.0]	0.164
Combined resection	7 (7.5%)	9 (5.5%)	1.000
Open surgery	47 (50.5%)	81 (50.0%)	1.000
**Anaesthesia duration, hour**	**3.0 [2.4; 3.7]**	**3.3 [2.6;4.3]**	**0.008**
**Surgical duration, hour**	**1.9 [1.5; 2.7]**	**2.3 [1.6; 3.2]**	**0.024**

Data are presented as the mean ± standard deviation median (interquartile range), or number (percentages).

BMI, body mass index; SBP, systolic blood pressure. *Fisher’s exact test was used for analysis.

## Discussion

Preoperative preparation, including the use of α-adrenergic receptor blockers to reduce intraoperative HI, is recommended in patients diagnosed with PPGL. If patients are undiagnosed and unprepared, the surgical treatment of PPGLs can lead to life-threatening complications (17). We determined the HI risk in PPGL patients whose diagnosis was preoperatively missed, probably due to no or mild symptoms and atypical locations. Although the mean SBP was higher in postoperatively diagnosed patients than in preoperatively diagnosed patients, there were no significant differences in the prevalence of intraoperative hypertension between the two groups, and there was a lower prevalence of intraoperative hypotension in postoperatively diagnosed PPGL patients. In addition, we suggest pheochromocytoma, maximal tumour size, and surgical duration as risk factors for intraoperative hypotension, which should be considered as a major component of intraoperative HI.

Despite advances in diagnosis and tumour localization methods, some PPGLs were detected intraoperatively due to an abruptly high BP or postoperatively after the histopathologic evaluation of surgically retrieved specimens. In the present study, 11.3% (29/256) of PPGL patients were diagnosed postoperatively. In postoperatively diagnosed PPGL patients, surgeries were performed based on an imaging diagnosis of retroperitoneal or pancreatic tumours. These results are similar to those from a recent study (11). Thomson et al. (11) analysed perioperative characteristics and clinical outcomes, including surgical complications, in 551 patients with pheochromocytoma who underwent surgery using Eurocrine^®^, a pan-European quality registry for endocrine tumours, from January 2015 to March 2020. They reported that 11.8% of patients underwent surgery due to suspected malignancy or tumour size and were diagnosed with pheochromocytoma postoperatively. There were no significant differences in postoperative complications between preoperatively diagnosed and postoperatively diagnosed patients.

In the present study, we focused on clinical characteristics and preoperative and intraoperative haemodynamic variables. As preoperative parameters, hypertension, pheochromocytoma, use of α-blockers, and β-blockers were significantly higher in preoperatively diagnosed PPGL patients than in postoperatively diagnosed PPGL patients. Regarding intraoperative haemodynamic parameters, postoperatively diagnosed PPGL patients had higher BP and HR, but lower intraoperative hypotension than preoperatively diagnosed PPGL patients. Mild catecholamine secretion and less use of α-blockers and β-blockers in postoperatively diagnosed patients may be attributed to the low incidence of intraoperative hypotension. However, since biochemical tests were not performed in postoperatively diagnosed patients, the severity of catecholamine excess cannot be assessed. In the present study, there was no 30-day mortality related to surgery in either group. A lower proportion of pheochromocytoma (17.2%) in postoperatively diagnosed PPGL patients and advances in surgical and anaesthesia techniques may have influenced the perioperative outcomes, including the duration of hospitalization, readmission rate, and 30-day surgery-related mortality. In the subgroup analysis, we also explored the haemodynamic parameters of only patients with paraganglioma. The intraoperative HI did not differ between the two groups except for a slightly higher mean MBP in postoperatively diagnosed patients. Therefore, regardless of tumour subtype, intraoperative hypertension might be related to intrinsic tumour features rather than the use of α-blockers.

Several studies have investigated potential predictive factors for intraoperative HI using different definitions of HI (6, 7, 12, 13, 18). There were discrepancies in predictive factors for intraoperative HI in each study. Kiernan et al. (12) evaluated HI as the number of intraoperative episodes of SBP >200 mmHg, those greater than or less than 30% of baseline, HR>100 bpm, and the need for postoperative vasopressors and reported that tumour size, open adrenalectomy, and type of α blockade were associated with intraoperative HI. Jiang et al. (7) defined intraoperative HI as the presence of at least one intraoperative SBP>200 mmHg episode and/or at least one intraoperative episode of DBP<80 mmHg and reported that tumour size, diabetes/prediabetes, and preoperative SBP fluctuation >50 mmHg were significant predictors for intraoperative HI. Ma et al. (18) defined HI as more than 10 episodes of SBP above 30% baseline or DBP less than 30% baseline despite vasoactive drug administration and reported that large tumour size and increased levels of urinary epinephrines were predictors of HI.

The strength of the present study is that we considered time to evaluate intraoperative HI quantitively and used several HI parameters, including time of SBP>160 mmHg (min), time of SBP>160 mmHg (%), time of MBP<60 mmHg (min), time of MBP<60 mmHg (%), time of HR>100 (min), time of HR>100 (%), ARV, MDPE, MDAPE, and Wobble. ARV, MDPE, MDAPE, and Wobble have been used as reliable and quantitative measures to evaluate BP variability in previous studies (14–16). Even though we assessed HI using various HI parameters, the incidence of intraoperative HI, except time of MBP<60 mmHg (min) and time of MBP<60 mmHg (%), was not different between groups. This may be because most patients were intensively monitored under continuous BP monitoring and were immediately treated using fast-acting intravenous vasodilators such as nicardipine.

We also analysed the incidence and risk factors for intraoperative hypotension (MBP <60 mmHg) due to a higher proportion of intraoperative hypotension cases included in intraoperative HI. Intraoperative hypotension was present in 63.7% (163/256) of PPGL patients, and pheochromocytoma, maximal tumour size, and surgical duration were significantly associated with intraoperative hypotension in PPGL patients. Several studies have reported an association between intraoperative hypotension and postoperative complications, including myocardial injury, acute kidney injury, stroke, and death, in patients undergoing cardiac and noncardiac surgeries (19–21). However, there is little information on predictive factors for intraoperative hypotension in PPGL patients. Gaujoux et al. (6) defined perioperative HI as the need for a cumulative perioperative dose of >5 mg norepinephrine and reported that symptomatic preoperative high BP and a tenfold increase in urinary metanephrine and/or normetanephrine levels were significant predictors for HI. Li et al. (22) categorized intraoperative hypotension into SBP ≤ 100, 95, 90, 85, and 80 mmHg with durations of 1, 5, 10, 20, 30, 40, and 50 min and reported that intraoperative hypotension was associated with an increased risk of postoperative complications. They suggested a dose-dependent relationship of harmful effects of intraoperative hypotension (22). Based on the present study, patients with pheochromocytoma, large tumours, and prolonged surgical duration should be treated careful to prevent intraoperative hypotension and postoperative complications.

The present study has several limitations. First, this is a retrospective study, and selective bias was not avoidable. Second, we could not reveal precise reasons for the lack of a significant difference in the prevalence of intraoperative hypertension and the lower prevalence of intraoperative hypotension in postoperatively diagnosed PPGL patients because the preoperative levels of serum and urine catecholamine in postoperatively diagnosed PPGLs were not available.

## Conclusions

Our study indicates that postoperatively diagnosed PPGLs do not have a higher risk of intraoperative hypertension and have a lower intraoperative hypotension risk than preoperatively diagnosed PPGLs. Intrinsic tumour characteristics such as pheochromocytoma, maximal tumour size, and intraoperative management might be more related to intraoperative HI than α-blocker use. Despite the limitations of retrospective studies, the present investigation provides valuable information for endocrine surgeons, endocrinologists, and anaesthesiologists to manage PPGL patients preoperatively and intraoperatively considering the high incidence of HI and risk factors for intraoperative HI and intraoperative hypotension.

## Data Availability Statement

The original contributions presented in the study are included in the article. Further inquiries can be directed to the corresponding author.

## Ethics Statement

The studies involving human participants were reviewed and approved by Institutional Review Board of the Seoul National University Hospital (IRB No. H-1801-010-911). Written informed consent for participation was not required for this study in accordance with the national legislation and the institutional requirements.

## Author Contributions

JHK: conceptualization, visualization, and writing-original draft. H-CL: formal analysis and writing-original draft. S-JK: conceptualization, writing-original draft, and supervision. KEL: investigation and writing - review and editing. KCJ: investigation and writing - review and editing. All authors contributed the article and approved the submitted version.

## Funding

This work was supported by a National Research Foundation of Korea (NRF) grant funded by the Korean government (MSIP) (grant numbers: 2018R1C1B5045216 and 2021R1F1A1055710).

## Conflict of Interest

The authors declare that the research was conducted in the absence of any commercial or financial relationships that could be construed as a potential conflict of interest.

## Publisher’s Note

All claims expressed in this article are solely those of the authors and do not necessarily represent those of their affiliated organizations, or those of the publisher, the editors and the reviewers. Any product that may be evaluated in this article, or claim that may be made by its manufacturer, is not guaranteed or endorsed by the publisher.

## References

[1] Kim JH Moon H Noh J Lee J Kim SG. Epidemiology and Prognosis of Pheochromocytoma/Paraganglioma in Korea: A Nationwide Study Based on the National Health Insurance Service. Endocrinol Metab (2020) 35(1):157–64. doi: 10.3803/EnM.2020.35.1.157 PMC7090309 32207276

[2] Bravo EL. Pheochromocytoma: Current Perspectives in the Pathogenesis, Diagnosis, and Management. Arq Bras Endocrinol Metabol (2004) 48(5):746–50. doi: 10.1590/S0004-27302004000500021 15761546

[3] Prejbisz A Lenders JW Eisenhofer G Januszewicz A. Cardiovascular Manifestations of Phaeochromocytoma. J Hypertens (2011) 29(11):2049–60. doi: 10.1097/HJH.0b013e32834a4ce9 21826022

[4] Zelinka T Petrák O Turková H Holaj R Strauch B Kršek M. High Incidence of Cardiovascular Complications in Pheochromocytoma. Horm Metab Res (2012) 44(5):379–84. doi: 10.1055/s-0032-1306294 22517556

[5] Kinney MA Narr BJ Warner MA. Perioperative Management of Pheochromocytoma. J Cardiothorac Vasc Anesth (2002) 16(3):359–69. doi: 10.1053/jcan.2002.124150 12073213

[6] Gaujoux S Bonnet S Lentschener C Thillois JM Duboc D Bertherat J. Preoperative Risk Factors of Hemodynamic Instability During Laparoscopic Adrenalectomy for Pheochromocytoma. Surg Endosc (2016) 30(7):2984–93. doi: 10.1007/s00464-015-4587-x 26684206

[7] Jiang M Ding H Liang Y Tang J Lin Y Xiang K. Preoperative Risk Factors for Haemodynamic Instability During Pheochromocytoma Surgery in Chinese Patients. Clin Endocrinol (2018) 88(3):498–505. doi: 10.1111/cen.13544 29292527

[8] Ye YL Yuan XX Chen MK Dai YP Qin ZK Zheng FF. Management of Adrenal Incidentaloma: The Role of Adrenalectomy may be Underestimated. BMC Surg (2016) 16(1):41. doi: 10.1186/s12893-016-0154-1 27278528 PMC4898397

[9] Kopetschke R Slisko M Kilisli A Tuschy U Wallaschofski H Fassnacht M. Frequent Incidental Discovery of Phaeochromocytoma: Data From a German Cohort of 201 Phaeochromocytoma. Eur J Endocrinol (2009) 161(2):355–61. doi: 10.1530/EJE-09-0384 19497985

[10] Gruber LM Hartman RP Thompson GB McKenzie TJ Lyden ML Dy BM. Pheochromocytoma Characteristics and Behavior Differ Depending on Method of Discovery. J Clin Endocrinol Metab (2019) 104(5):1386–93. doi: 10.1210/jc.2018-01707 30462226

[11] Thompson LH Makay Ö Brunaud L Raffaelli M Bergenfelz A. Adrenalectomy for Incidental and Symptomatic Phaeochromocytoma: Retrospective Multicentre Study Based on the Eurocrine^®^ Database. Br J Surg (2021) 108(10):1199–206. doi: 10.1093/bjs/znab199 PMC10364866 34270711

[12] Kiernan CM Du L Chen X Broome JT Shi C Peters MF. Predictors of Hemodynamic Instability During Surgery for Pheochromocytoma. Ann Surg Oncol (2014) 21(12):3865–71. doi: 10.1245/s10434-014-3847-7 PMC4192065 24939623

[13] Kim JH Lee HC Kim SJ Yoon SB Kong SH Yu HW. Perioperative Hemodynamic Instability in Pheochromocytoma and Sympathetic Paraganglioma Patients. Sci Rep (2021) 11(1):18574. doi: 10.1038/s41598-021-97964-3 34535733 PMC8448751

[14] Mena L Pintos S Queipo NV Aizpurua JA Maestre G Sulbaran T. A Reliable Index for the Prognostic Significance of Blood Pressure Variability. J Hypertens (2005) 23(3):505–11. doi: 10.1097/01.hjh.0000160205.81652.5a 15716690

[15] Lee HC Ryu HG Jung CW. Performance Measurement of Intraoperative Systolic Arterial Pressure to Predict in-Hospital Mortality in Adult Liver Transplantation. Sci Rep (2017) 7(1):7030. doi: 10.1038/s41598-017-07664-0 28765633 PMC5539171

[16] Mascha EJ Yang D Weiss S Sessler DI. Intraoperative Mean Arterial Pressure Variability and 30-Day Mortality in Patients Having Noncardiac Surgery. Anesthesiology (2015) 123(1):79–91. doi: 10.1097/ALN.0000000000000686 25929547

[17] Sutton MG Sheps SG Lie JT. Prevalence of Clinically Unsuspected Pheochromocytoma. Review of a 50-Year Autopsy Series. Mayo Clin Proc (1981) 56(6):354–60. 6453259

[18] Ma L Shen L Zhang X Huang Y. Predictors of Hemodynamic Instability in Patients With Pheochromocytoma and Paraganglioma. J Surg Oncol (2020) 122(4):803–8. doi: 10.1002/jso.26079 PMC7496938 32562589

[19] Salmasi V Maheshwari K Yang D Mascha EJ Singh A Sessler DI. Relationship Between Intraoperative Hypotension, Defined by Either Reduction From Baseline or Absolute Thresholds, and Acute Kidney and Myocardial Injury After Noncardiac Surgery: A Retrospective Cohort Analysis. Anesthesiology (2017) 126(1):47–65. doi: 10.1097/ALN.0000000000001432 27792044

[20] Sun LY Chung AM Farkouh ME van Diepen S Weinberger J Bourke M. Defining an Intraoperative Hypotension Threshold in Association With Stroke in Cardiac Surgery. Anesthesiology (2018) 129(3):440–7. doi: 10.1097/ALN.0000000000002298 29889106

[21] Monk TG Bronsert MR Henderson WG Mangione MP Sum-Ping ST Bentt DR. Association Between Intraoperative Hypotension and Hypertension and 30-Day Postoperative Mortality in Noncardiac Surgery. Anesthesiology (2015) 123(2):307–19. doi: 10.1097/ALN.0000000000000756 26083768

[22] Li N Kong H Li SL Zhu SN Zhang Z Wang DX. Intraoperative Hypotension Is Associated With Increased Postoperative Complications in Patients Undergoing Surgery for Pheochromocytoma-Paraganglioma: A Retrospective Cohort Study. BMC Anesthesiol (2020) 20(1):147. doi: 10.1186/s12871-020-01066-y 32532209 PMC7291712

